# Supplementary material for: A comparison of long-term maternal mortality associated with pathologic placental separation: Highlighting possible trends and mechanisms
Source: PLoS One. 2026 Apr 24;21(4):e0338586. doi: 10.1371/journal.pone.0338586 (PMC13108790; doi:10.1371/journal.pone.0338586)
Supplement: S1 Table — (DOCX) [file pone.0338586.s001.docx]

**S1 Appendix Table of placental outcome ICD codes**

| **Condition** | **ICD-10 Codes** | **ICD-9 Codes** |
| --- | --- | --- |
| Placental Abruption | O45, O45.0, O45.00, O45.01, O45.02, O45.09, O45.001, O45.002, O45.003, O45.009, O45.011, O45.012, O45.013, O45.019, O45.021, O45.022, O45.023, O45.029, O45.091, O45.092, O45.093, O45.099, O45.8, O45.8X, O45.8X1, O45.8X2, O45.8X3, O45.8X9, O45.9, O45.90, O45.91, O45.92, O45.93 | 641.31, 641.33, 641.2, 641.20, 641.21, 641.23 |
| Retained Placenta | O73, O73.0, O73.1 | 667, 667.1, 667.10, 667.12, 667.14, 667.0, 667.00, 667.02, 667.04 |
