# Supplementary material for: A comparison of long-term maternal mortality associated with pathologic placental separation: Highlighting possible trends and mechanisms
Source: PLoS One. 2026 Apr 24;21(4):e0338586. doi: 10.1371/journal.pone.0338586 (PMC13108790; doi:10.1371/journal.pone.0338586)
Supplement: Attachment — Submitted filename: Response_to_Reviewers_PLOS_ONE-2.pdf [file pone.0338586.s009.pdf]

Response to the Editor and Reviewers

Manuscript title: A comparison of long-term maternal mortality associated with pathologic placental separation: highlighting possible trends and mechanisms

Dear Editor and Reviewers,

We sincerely thank you for the time and expertise you devoted to reviewing our manuscript. We greatly value the constructive feedback and have undertaken a comprehensive revision that strengthens the clarity, methodologic transparency, and interpretive rigor of the study. Throughout, we have revised the manuscript to ensure that conclusions remain strictly supported by the data, and we have clarified when analyses are descriptive/associational rather than causal.

Summary of major revisions

- Clarified scope and terminology: revised the title and abstract to specify "long-term maternal mortality," and reduced causal language throughout.
- Improved reproducibility: added explicit ICD-9-CM and ICD-10-CM code definitions for placental abruption and retained placenta, and specified the temporal window used to link diagnostic codes to the index delivery; full code lists are provided in Supporting Information (S1 Appendix).
- Reorganized results presentation: reworked Tables 1–3 to improve readability, to present absolute event counts and rates, and to clearly distinguish baseline characteristics/acute clinical interventions from broader associated diagnoses.
- Strengthened analytic transparency: clarified the Cox model specification and covariate strategy using a DAG-informed approach (primary adjustment set limited to demographic confounders) and added a secondary model that adjusts for selected pre-existing conditions.
- Addressed proportional hazards concerns: evaluated proportional hazards using Schoenfeld residuals (global and variable-specific) and added complementary analyses including time-window–restricted models and piecewise Cox models (Supporting tables).
- Addressed sparse-data and multiplicity concerns: reported events-per-variable for core mortality models (Supporting Information) and applied Bonferroni and Benjamini–Hochberg corrections for primary mortality comparisons; we also explicitly frame secondary associations as exploratory.
- Expanded and tempered interpretation: clarified limitations related to ICD-based definitions, temporality of "health condition" associations, and incomplete BMI data; refined conclusions and clinical implications accordingly.
- Improved writing and formatting: performed thorough proofreading and standardized references/citations to journal requirements.

Point-by-point responses

We thank the reviewers for their thoughtful and constructive comments. We have carefully addressed each point and made substantial revisions to strengthen the manuscript. Below we provide a detailed, point-by-point response to each reviewer. For ease of review, all edits are visible in the revised manuscript with highlighted texts, and we reference key updated tables/appendices where applicable.

Reviewer #1

Comment: *The presentation of the copious amount of data within the manuscript's tables is difficult to understand; please rework tables and explain how data were handled.*

Response: We sincerely thank you for the careful and constructive evaluation of our manuscript. We have completed a substantial revision to address your comments and concerns. We agree and have comprehensively restructured Tables 1–3 and expanded the Results narrative to guide readers through the key findings. Specifically: (i) Table 1 is now a clearly labeled baseline characteristics table that additionally reports follow-up time, person-years, and absolute death counts/rates to contextualize subsequent survival analyses; (ii) Table 2 now presents the core mortality hazard ratios with clearly defined adjustment sets and complementary sensitivity analyses to separate short-term vs long-term patterns; (iii) Table 3 has been reframed as a set of adjusted odds ratios from multivariable logistic regression to describe associated diagnoses and we added explicit criteria for how "comparative directionality" is defined. We also expanded the Methods to clarify model specification, covariate selection, and assumption-checking.

Comment: *Table 1: It is unclear what the p value refers to; with very different group sizes, statistics seem superfluous. Also, why report year of birth; maternal age would be better.*

Response: Thank you—this was an important clarity issue. We revised Table 1 to remove ambiguity and improve clinical interpretability. First, we removed p-values for the cohort size and other descriptive rows where hypothesis testing is not informative; where p-values remain, they now correspond to clearly specified overall group comparisons for that row, and the table header/footnote explains the tests used. Second, we added maternal age at delivery (mean, SD) as a primary demographic descriptor and use it in the primary adjusted mortality model. We also added absolute death counts and mortality rates (per 1,000 deliveries and per 1,000 person-years) to facilitate within-group interpretation rather than relying on significance testing alone.

Comment: *SDOH is qualitative; what do the numbers represent, and how does statistical evaluation help?*

Response: We thank the reviewer for this comment. In this study, social determinants of health (SDOH) were identified using ICD-10-CM–based social risk indicator codes available in the TriNetX research network. We operationalized SDOH as **binary variables** (presence vs absence of documented social risk), and the values shown therefore represent the **number (%) of patients with at least one recorded SDOH code** in each category/group rather than a graded or qualitative measure of social context. We then evaluated whether these *coded* SDOH indicators, as defined within TriNetX, differed across abnormal placental separation groups and were associated with long-term mortality in adjusted analyses. We recognize that ICD-coded SDOH captures only the portion of social risk that is documented in the medical record and does not reflect the full breadth or nuance of SDOH. To address this, we clarified in the **Methods** and **Table 1** that SDOH in TriNetX is based on ICD-10-CM social risk indicator codes and is analyzed as a binary indicator (presence/absence of any documented social risk code). We also clarified that SDOH is included as a demographic confounder in our primary adjusted mortality model to account for potential differences in documented social risk burden across groups.

Comment: *Some entries listed under Health Outcomes are not outcomes (alcohol/tobacco; procedures like transfusion; multiple gestation).*

Response: We agree and revised both the labeling and analytic framing. In Table 1, alcohol/tobacco and acute interventions (e.g., transfusion, critical care) are now presented as baseline characteristics and acute clinical factors recorded around the delivery encounter rather than as "health outcomes." In Table 3, we refer to the broader set of diagnoses as "associated health conditions," and we explicitly note that, due to database limitations, these associations may reflect conditions present before or after the index delivery. Accordingly, we interpret Table 3 as exploratory associations that may inform hypotheses for future mechanistic and prospective work, rather than as causal or incident outcomes.

Comment: *Table 2: Please specify the formula used to determine hazard ratios and how adjustment factors were identified and used.*

Response: We expanded the Statistical Analysis section to explicitly describe the Cox proportional hazards regression framework and the interpretation of the hazard ratio as a relative risk of death over follow-up. We also clarified covariate selection using a DAG-informed approach to avoid overadjustment for potential mediators/colliders. Our primary adjusted model controls for demographic confounders (maternal age at delivery, race, and SDOH indicator). A secondary model adds selected pre-existing conditions (diabetes, hypertension, obesity, chronic kidney disease) to evaluate robustness to baseline health status. In addition, Table 2 now includes complementary analyses (censoring at 1 year; excluding deaths ≤42

days; excluding deaths ≤1 year; and piecewise Cox models in Supporting Information) to better characterize temporal patterns.

Comment: *Table 3 directionality is opaque; some numerically different values are called "similar," while closer values are "different." Discussion does little to clarify inconsistencies.*

Response: Thank you for highlighting this. We revised Table 3 in three ways: (i) We changed the analysis from Cox models to multivariable logistic regression and now report adjusted odds ratios (ORs), which is more appropriate for cross-sectional diagnosis associations; (ii) We added a clear definition for the "Comparative Directionality" column: "Similar" indicates both abruption and retention show statistically significant elevated association ($p<0.05$) in the same direction, whereas "Different" indicates statistical significance for only one condition; (iii) We revised the Results and Discussion to summarize the most clinically salient patterns and to avoid overinterpreting magnitude differences when the aim is to contrast broad association patterns between the two placental conditions.

Reviewer #2

Comment: *Some covariates in fully adjusted models could be downstream conditions; please clarify overadjustment potential and implications.*

Response: We sincerely thank you for the careful and constructive evaluation of our manuscript. We have completed a substantial revision to address your comments and concerns. We agree. In the original submission, the "fully adjusted" mortality model included a large number of health conditions that could plausibly lie on the causal pathway or reflect post-exposure diagnoses, which could introduce overadjustment and/or collider bias. In the revision, we adopted a DAG-informed strategy and defined a primary adjusted model limited to demographic confounders (maternal age at delivery, race, SDOH indicator). We deliberately avoided adjusting for acute delivery complications and downstream diagnoses in the primary mortality model. We retained a secondary model that adjusts for a small set of pre-existing conditions as a robustness check (not as a causal estimand) and we revised the Discussion to explicitly state that our findings are associational and do not establish causality.

Reviewer #3

Comment: *Revise "long-term mortality" to "long-term maternal mortality" in title and abstract.*

Response: We sincerely thank you for the careful and constructive evaluation of our manuscript. We have completed a substantial revision to address your comments and concerns. We revised both the title and the abstract to explicitly reference "long-term maternal mortality," improving immediate clarity for readers.

Comment: *Explicitly state ICD codes used to define placental abruption and retained placenta in the Methods.*

Response: We added ICD-9-CM and ICD-10-CM code families for placental abruption (ICD-9-CM 641.2x; ICD-10-CM O45.x) and retained placenta (ICD-9-CM 667.0x–667.1x; ICD-10-CM O72.0–O72.2). We also clarified the temporal linkage: placental-separation codes occurring within 6 weeks before to 6 weeks after the delivery date were used to classify the index delivery. Full code lists are provided in Supporting Information (S1 Appendix).

Comment: *Expand interpretation of age-related findings in the Discussion.*

Response: We expanded the Discussion to contextualize the observed age differences and to emphasize maternal age as a key determinant of baseline health and obstetric risk. We also incorporate maternal age directly into the primary adjusted mortality model and discuss how age-related confounding may contribute to long-term mortality differences.

Reviewer #4

Comment: *1. Conceptual and causal overreach; mechanistic speculation without sufficient justification; inappropriate adjustment for post-exposure variables and interpreting residual associations as causal.*

Response: We sincerely thank you for the careful and constructive evaluation of our manuscript. We have completed a substantial revision to address your comments and concerns. We agree with the need to avoid causal overinterpretation. In the revised manuscript, we substantially reduced causal language and reframed mechanistic discussion as hypothesis-generating rather than confirmatory. Critically, we revised the mortality modeling strategy to avoid adjusting for downstream diagnoses/complications in the primary model. We now emphasize that the study is observational and that temporality cannot be fully established for many associated diagnoses in the database. Accordingly, we interpret mortality associations as risk markers and interpret diagnosis associations (Table 3) as exploratory correlates that may inform future mechanistic and prospective validation studies.

Comment: *2. Inappropriate adjustment strategy; lack of DAG or conceptual framework.*

Response: We added a DAG-informed covariate selection rationale in the Methods. Our primary adjusted model includes only demographic confounders (maternal age at delivery, race, SDOH indicator). We explicitly avoided adjusting for acute delivery complications (e.g., hemorrhage/DIC) or downstream conditions in the primary mortality model. We include a secondary model with selected pre-existing conditions

to assess robustness to baseline health status, and we clarify that this is a sensitivity/robustness analysis rather than a causal adjustment for mediators.

Comment: *3. Proportional hazards assumption violated in adjusted models; suggested alternatives not explored; sensitivity analyses may change estimand.*

Response: We agree that proportional hazards assessment and appropriate alternatives are essential. In the revision, we report proportional hazards diagnostics using Schoenfeld residuals and provide both global and variable-specific tests. Importantly, variable-specific testing shows that **the exposure (placental separation group) satisfies the PH assumption**, while the global violation is driven by adjustment covariates. To further address potential time-varying effects, we added complementary analyses including (i) time-window–restricted models (short-term within 1 year; excluding ≤42-day deaths; excluding ≤1-year deaths) and (ii) piecewise Cox models across prespecified intervals (0–1, 1–2, 2–5, >5 years) presented in Supporting Information. We clarify that time-window analyses evaluate related but distinct estimands and therefore are presented as complementary descriptions of temporal risk patterns rather than "fixes" for the primary model.

Comment: *4. Sparse data bias/overfitting/multiple comparisons; missing event counts, EPV ratios, and correction for multiple testing.*

Response: We addressed these concerns in multiple ways. First, we now report absolute death counts by group, follow-up time, and person-years (Table 1 and Results). Second, we quantified events-per-variable (EPV) for core mortality models and demonstrate that EPV is very high for the primary mortality analyses (Supporting Information), mitigating overfitting concerns for those models. Third, for primary mortality outcomes we applied Bonferroni and Benjamini–Hochberg FDR corrections and report corrected inference in Supporting Information. Finally, we explicitly label the broader diagnosis associations (Table 3) as exploratory and interpret them cautiously, emphasizing pattern recognition rather than isolated p-values for rare diagnoses.

Comment: *5. Reliance on administrative codes without validation; misclassification concerns and potential differential coding.*

Response: We agree that ICD-based phenotyping is a key limitation. We strengthened the Methods by providing explicit code definitions and the temporal window used to link diagnosis codes to the delivery encounter. We also expanded the Limitations to discuss misclassification risk and cite evidence that prevalence estimates can differ between ICD-based definitions and clinical review. We acknowledge that coding inaccuracies and care-intensity–dependent documentation could introduce bias (including differential misclassification) and that the direction of

such bias is not guaranteed. We therefore frame our findings as associations that warrant validation in datasets with chart adjudication or prospectively defined clinical phenotypes.

Comment: *6. Ambiguity in temporality of "health outcomes"; Table 3 conflates baseline risk factors, acute complications, and long-term sequelae.*

Response: We agree, and we revised both the terminology and interpretation. In the revised manuscript, we refer to the non-mortality diagnoses as "associated health conditions" rather than "outcomes". We also separated the mortality analyses (time-to-event) from the diagnosis association analyses (cross-sectional logistic regression) and revised the Discussion and Limitations to emphasize that these associations do not establish chronology.

Comment: *7. Inconsistent/confusing interpretation of obesity; incomplete BMI/pregnancy weight gain data.*

Response: We clarified this issue in the revised Limitations. We report obesity as captured in TriNetX and acknowledge incomplete BMI/weight-gain data. We therefore interpret obesity findings cautiously and present them as potentially reflecting differences in coding completeness, baseline risk profiles, and unmeasured confounding. We also included obesity only in the secondary "pre-existing conditions" model as a robustness check rather than as a definitive explanatory factor.

Comment: *8. Overstated clinical implications; conclusions should be strictly supported by the data.*

Response: We agree and have substantially tempered the clinical implications. We revised the Discussion and Conclusion to avoid prescriptive recommendations for postpartum surveillance based solely on these observational associations. The revised Conclusion now emphasizes that abnormal placental separation is associated with elevated long-term maternal mortality after demographic adjustment, that the temporal pattern differs between abruption and retention, and that further research is needed to clarify mechanisms and to validate clinically actionable risk stratification.

Comment: *Minor concerns: grammar, punctuation, citation formatting, and table summarization.*

Response: We carefully proofread the entire manuscript, corrected grammatical and typographical issues, improved transitions, and ensured that key table findings are summarized in the text. We also standardized reference formatting and updated incomplete citations to align with journal requirements.

We again thank the editor and reviewers for the thoughtful feedback. We believe these revisions have substantially strengthened the manuscript’s clarity, methodological transparency, and interpretability, and we hope it is now suitable for publication.

Sincerely,

On behalf of all authors

Atalay Demiray, MD, MSc

Atalay Demiray, MD
